# Evidence map of appendicitis – a living systematic review with meta-analyses

**DOI:** 10.1007/s00423-026-04113-3

**Published:** 2026-07-01

**Authors:** Dirk Kleindienst, Justyna Mohr, Katharina Maurer, Eva Kalkum, Julian-Camill Harnoss, Pia Antony, Alexander Dullenkopf, Pietro Renzulli, Pascal Probst, Markus K. Mueller

**Affiliations:** 1 Department of Surgery, Cantonal Hospital Thurgau, Münsterlingen, Switzerland; 2 Study Center of the German Society of Surgery (SDGC), University of Heidelberg, Heidelberg, Germany; 3 Department of Surgery, Cantonal Hospital Thurgau, Frauenfeld, Switzerland

**Keywords:** Evidence map, Appendicitis, Surgery, Systematic review, Meta-analysis, Living review

## Abstract

**Purpose:**

Appendicitis is one of the most common diseases of the gastrointestinal tract with a lifetime incidence of 7 to 9%. Appendectomy is one of the most performed emergency surgeries. Recently, non-operative treatments are emerging. It is almost impossible to keep the overview on the large and growing body of evidence on appendicitis. Therefore, the aim of this project was to create a systematic and living Evidence Map of Appendicitis.

**Methods:**

PubMed, CENTRAL, Web of Science, EMBASE were systematically and continuously searched for all published and ongoing randomized controlled trials (RCT) and systematic reviews (SR) dealing with diagnosis and treatment of appendicitis. Outcomes from every existing RCT were extracted and trial quality was assessed. RCTs and SRs on identical subjects were grouped to research topics. For specific treatment outcomes, meta-analyses were performed for all research topics with more than 3 RCTs.

**Results:**

Out of 16373 articles screened, 527 RCTs on 104331 patients and 408 SRs were included and grouped into 74 research topics with 31 living meta-analyses. Most RCTs were from Asia (43%),followed by Europe (32%) and North America (15%). Living meta-analyses were performed for 31 out of 74 research topics. For three out of 74 research topics (4%) there were no RCT available.

**Conclusion:**

The Evidence Map of Appendicitis is the ultimate go-to source for evidence on appendicitis. Constantly updated and freely accessible via www.EVIglance.com and as mobile phone app. Clinical decision-making, evidence-based patient information are supported by the primary data provided, as well as by living meta-analyses.

**Systematic review registration:**

PROSPERO 2023 CRD42023482686.

**Supplementary Information:**

The online version contains supplementary material available at https://doi.org/10.1007/s00423-026-04113-3.

## Background

The first open appendectomy was described by McBurney in 1889 [1, 2]. Due to efficacy and safety this procedure evolved into the preferred standard treatment in case of acute appendicitis more than one century. Semm performed the first laparoscopic appendectomy in 1980 [3, 4]. According to technical improvements of the laparoscopic technique, their association with lower incidence of wound infection, lower post-intervention morbidity, shorter hospital stay and better quality of life scores compared to open appendectomy, the laparoscopic treatment became standard of care over the last decades [3, 5, 6]. Nowadays, possibilities of non-operative treatment strategies are in the focus of appendicitis research [7–9]. Ongoing discussions address the timing, duration, and route of antibiotic administration, particularly in complicated appendicitis and perityphlitic abscess management [10, 11].

The rapidly expanding body of literature on appendicitis, spanning diagnostic innovations and evolving therapeutic strategies, demands continuous, structured synthesis and critical appraisal. Yet such an overview is currently lacking, leaving physicians and researchers inundated with a daily stream of new, largely unsorted publications.Therefore, the aim of this project was to create a systematic and living Evidence Map of Appendicitis, providing physicians with swift, intuitive, and perpetual access to evidence.

## Methods

This systematic review is reported according to PRISMA-LSR guidelines [12], where applicable. The project was prospectively registered (PROSPERO 2023 CRD42023482686). This paper-based publication is the current and citable version of the Evidence Map of Appendicitis. However, the map content itself will be developed beyond that of the current version that this manuscript describes.

Systematic reviews produce highly reliable findings of all available literature research of one topic at one timepoint. A “living systematic review” instead is intended to deliver ongoing “living” updates of the research content by recurrent ongoing updates of the map content in the future.

The reviewers completed each aspect of the study process, including screening, data extraction, analysis and creation of the map. The project itself is funded by Lexington Medical, however, there was no influence of the content and creating process of the map.

### Systematic literature search

A systematic search of all major electronic bibliographic databases relevant for surgery was performed [13]: MEDLINE (via PubMed), Web of Science, and Cochrane Central Register of Controlled Trials (CENTRAL). Additionally, EMBASE was searched because non-surgical interventions were included as well. No restrictions were applied regarding language or publication date. The search strategy used a combination of key search terms as index or free text words and MeSH terms aiming to cover the whole field of operative and non-operative treatment strategies of appendicitis. The full search strategy for all databases can be found in appendix 1.The last search for Version 1 (V1) was performed on June 12th 2026 [was freshly updated in order to publication].

### Study selection

All patients (adults and pediatric) with any kind of intervention due to appendicitis whether complicated or uncomplicated were considered eligible. Surgical interventions or non-operative treatment strategies that aimed to affect outcome of appendicitis were included. Also, diagnostic tools (e.g. clinical assessment, computer tomography, ultrasound or laboratory values) were investigated. Comparisons of operative treatments (e.g. open versus laparoscopic access to the abdominal cavity or three port versus single incision technique) or trials on emergency vs. elective setting for interval appendectomy were included. Trials investigating the use of medical devices (e.g. stapler versus hem-o-loc clips and trials on antibiotic drug use were also deemed eligible. Furthermore, perioperative management (e.g. enhanced recovery protocols, nutrition of patients or pain management) were included. Interventions like endoscopic retrograde appendicitis therapy, radiologically guided punctures of perityphlitic abscesses or similar were only included when they were compared to a surgical intervention. Studies dealing with surgery with malignant or premalignant tumours of the appendix were excluded.

Other reasons for excluding full text articles has been duplicate records not automatically detected during title and abstract screening, congress presentations or exclusion of RCT protocols after publication of the RCT.

RCTs and SRs with or without meta-analysis (MA) were eligible for inclusion. SRs were only eligible if they met minimum quality requirements, i.e., the SR must include at least two established literature databases and provide a critical appraisal with validated tools, such as the Cochrane Collaboration tool for assessing risk of bias [14] for RCTs.

Following the recommendations of the Cochrane Collaboration [15], two reviewers independently screened the titles, abstracts, and full texts of the identified articles. A third reviewer resolved any disagreement between the two reviewers. Screening was done with EVIglance STUDIO.

### Data extraction

Three reviewers using predefined items carried out all stages of data extraction and quality assessment. Another reviewer verified the extraction of every article. A senior reviewer resolved any disagreement. Extraction and verification was done with EVIglance STUDIO.

The outcomes of interest were mortality, morbidity (if available, according to the Clavien-Dindo classification [16]), comprehensive complication index [17], surgical site infection, re-admission, re-intervention or re-operation, operation time or adverse events in the antibiotic treatment. Additional outcomes including pain, quality of life, length of hospital stay, absence of work and costs were also extracted.

### Risk of bias assessment

Furthermore, the methodological quality of included RCTs was assessed with the Cochrane Collaboration tool for assessing risk of bias 2.0 [14]. Five standard domains of bias were assessed: bias arising from the randomization process, bias due to deviations from intended interventions, bias due to missing outcome data, bias in measurement of the outcome and bias in selecting of the reported result. These domains were rated as “high risk of bias”, “low risk of bias” or “some concerns”. Finally, an overall risk of bias judgment was made. The overall risk of bias was considered “high risk of bias” if at least one domain had a “high risk of bias” or if there were “some concerns” in three or more domains. The overall risk of bias was “some concerns” if there were “some concerns” in at least one domain. The overall risk of bias was “low risk of bias” if all domains were rated as “low risk of bias”. The blinding of patients, surgeons, data collectors, outcome assessors, and data analysts was assessed as “blinded”, “not blinded” or “not reported” [18]. Furthermore, information on funding was recorded as “industry”, “independent” or “not reported” [19].

### Data synthesis

The Evidence Map of Appendicitis is freely accessible via the Internet on www.EVIglance.com. All included RCTs and SRs on the same research topics are grouped together, e.g. “closure of the appendicular stump” or “laparoscopic appendectomy versus open appendectomy in adult and children”. Existing SRs are displayed within the evidence map and are used to identify evidence gaps i.e. missing RCTs for a research topic. No quantitative data from SRs are extracted and no critical appraisal of SRs was performed. But showing the data dealing with diagnostic issues, at this time it is not possible to meta-analyse diagnostic studies in EVIglance STUDIO. Ongoing RCTs and unpublished terminated RCTs are also displayed within the evidence map; these will be replaced as results become available.

### Creation of the evidence map

A summary, a flow chart as well as a section containing definitions and guidelines on appendicitis, appear at the top of the evidence map. The map itself is configured as a mind map in a manner that is intuitive and hierarchically leads its reader from the top (Evidence Map of Appendicitis) via main topics (e.g. diagnostic or treatment) and subtopics (e.g. surgical strategies or non-operative treatments) to individual research topics (e.g. single incision vs. multiport for laparoscopic appendectomy). Moreover, to optimize the use of the map for mobile phones (via the EVIglance app), there is also a tabular view of the map.

Within a research topic, existing RCTs and SRs are plotted with the name of the first author and year of publication. For every study, the conclusion of the article, the citation and a direct link to the article on the journal homepage are provided. Processed data from RCTs are provided allowing further meta-analyses for other researchers. EVIglance STUDIO is built in a manner, that after the initial publication, the effort to keep the map “living” is very limited. Following the continuous search the databases, the evidence map is using, the relevant studies will screened and implemented in the map. This work will be partly done automatically or is the responsibility of the team’s ongoing academic activity.

### Quantitative analyses and GRADE

Living meta-analyses are performed for research topics if there were at least 3 RCT data sets for an outcome available. Dichotomous data were pooled in a Mantel–Haenszel model to estimate odds ratios and associated 95% confidence intervals. For continuous data, mean differences and associated 95% confidence intervals were calculated using an inverse-variance model. A random effects model was used due to general clinical heterogeneity of surgical trials. For dichotomous and continuous data an exact p-value was calculated. Statistical heterogeneity was evaluated via the I^2^ statistic. An I^2^ < 25% was considered low statistical heterogeneity and an I^2^ > 75% was considered high statistical heterogeneity. A funnel plots was created if more than 10 RCTs are available for one outcome. EVIglance STUDIO was used for statistical analysis.

Furthermore, for each outcome in the meta-analyses, the certainty of the evidence was rated using the GRADE system [20, 21]. This system includes limitations in the design from the risk of bias assessment as mentioned above, indirectness of evidence, unexplained heterogeneity or inconsistency of results, imprecision of results, and publication bias. Thus, the certainty of evidence was rated as “very low”, “low”, “moderate” or “high” for each outcome.

Furthermore, all RCTs investigating exclusively one type of operation were pooled by a meta-analysis of proportions to create benchmarks e.g. surgical site infection rate with a 95% confidence interval after laparoscopic appendectomy.

### Living systematic review and meta-analyses

The map will be updated at least every 3 months, according to the above-mentioned steps. Moreover, relevant new articles found by hand search or discovered via social media will be added immediately. All new RCTs and SRs will be added to the map under their respective research topics. The date of last update will be reported in the online map and on the flow chart. Meta-analyses will be renewed when new RCTs become available, thus resulting in living meta-analyses. For every meta-analysis, the date of calculation will be provided. At this point metaanalysis of diagnostic studies are not available.

## Results

**Fig. 1:**
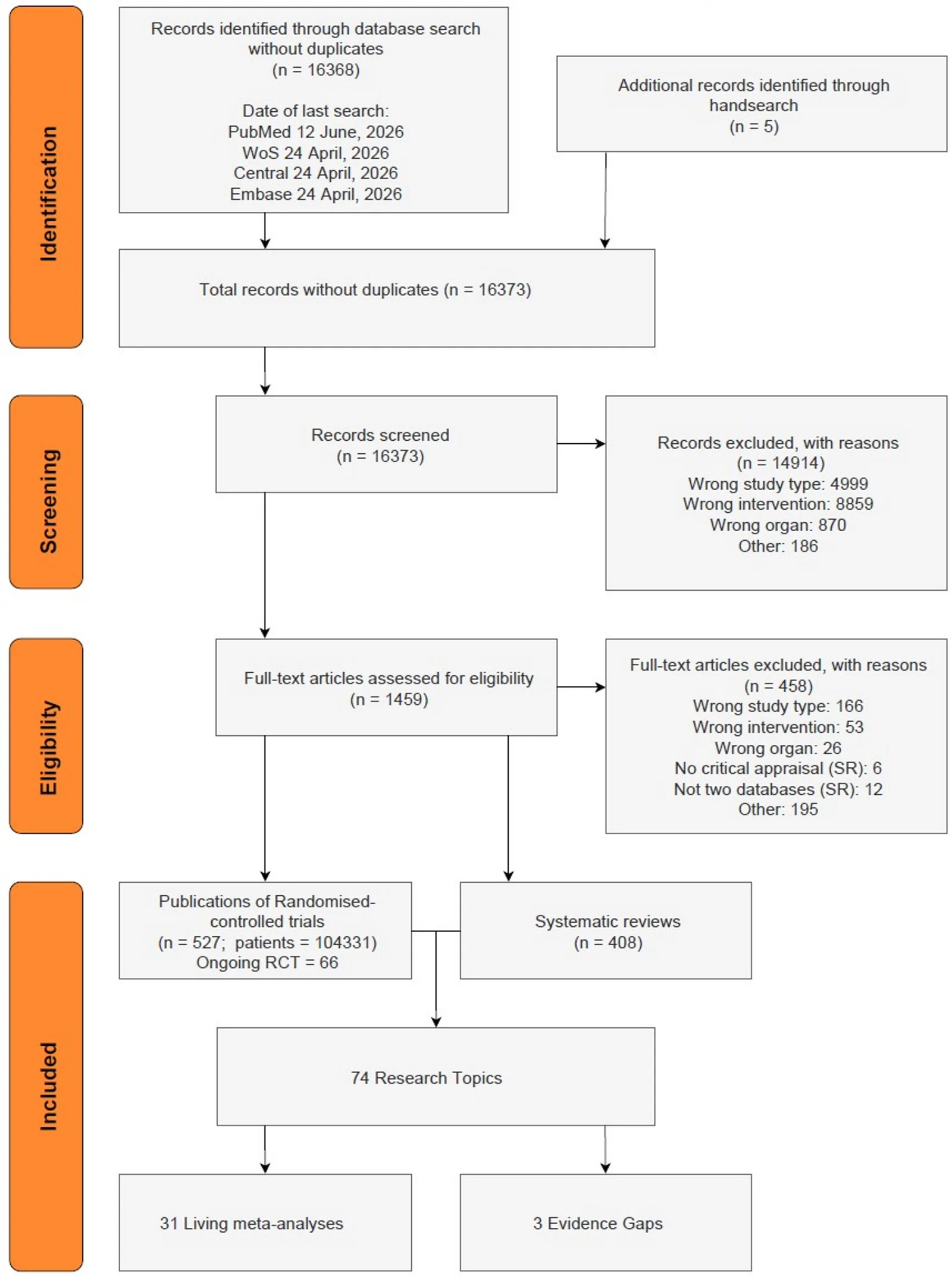
PRISMA flow chart

The Evidence Map of Appendicitis is freely accessible via www.EVIglance.com and the EVIglance app for android and iOS phones. This paper-based publication represents Version 1. Altogether, 16373 articles were identified via systematic literature search and 14877 articles were excluded after title and abstract screening. Of 1459 full-text articles assessed for eligibility, 527 published RCTs (including 104331 patients), 66 ongoing RCTs, and 408 SRs were included. Figure 1 shows the PRISMA flow chart of this process.

**Fig. 2:**
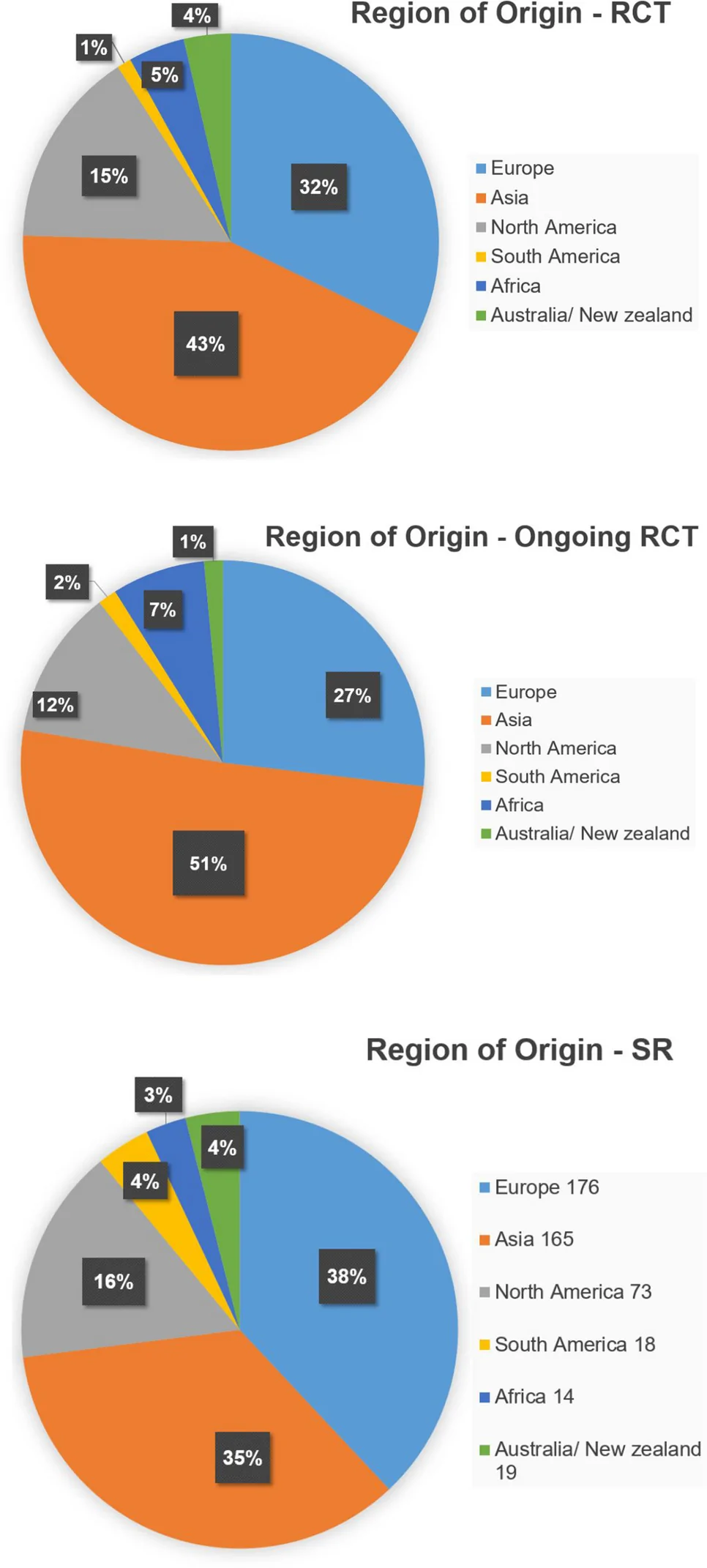
RCT: Origin of included RCT. Ongoing RCT: Origin of included ongoing RCT. SR: Origin of included SR

From 527 RCTs, twenty were performed multiregional resulting in a denominator of 5427 for the following calculations: The highest number of RCTs has, so far, been conducted in Asia (43%; 237 of 547), followed by Europe (32%; 176 of 547) and North America (15%; 84 of 547). Other geographical regions—Africa, Australia and New Zealand, and South America—contributed between 1 to 4% (Fig. 2 - RCT). In addition there are 66 RCTs ongoing. Most ongoing RCTs are running in Asia (51%; 34 of 66 ongoing RCTs) and Europe (27%; 18 of 66 ongoing RCTs), followed by North America (12%; 8 of 66 ongoing RCTs) and Africa (7%; 5 of 66 ongoing RCTs). Only 1% for each are contributed by South America and Australia/New Zealand (Fig. 2 – Ongoing RCT). A total of 58 SRs were performed were performed multiregional resulting in a denominator of 408 for the following calculations: The most SRs were conducted in Europe (38%; 176 of 408), followed by Asia (35%; 165 of 408), North America (16%; 73 of 408).

Australia/New Zealand (4%; 19 of 408), South America (4%; 18 of 408) and Africa (3%; 14 of 408) contributed together only 11% (Fig. 2 - SR).

### Map content

The current map consists of two main topics: “Diagnostics” and “Treatment”. Furthermore, “Definitions and Guidelines” comprises information of existing guidelines on appendicitis. The main topic, “Diagnostic”, contains four subtopics (“Scores”, “Laboratory values”, “Management”, and “Imaging”). The main topic “Treatment” contains three subtopics: “Non-operative strategies”, “Surgical strategies”, and “Pain and perioperative management.” In total, there are 74 research topics and for 31 out of 74 research topics (42%) living meta-analyses were performed and are available online via www.EVIglance.com. There was an evidence gap (i.e. lack of an RCT) for the “treatment” research topic “robotic appendectomy”. Table 1 gives a tabular overview on the research topics and if a living meta-analysis was conducted.

**Table 1:**
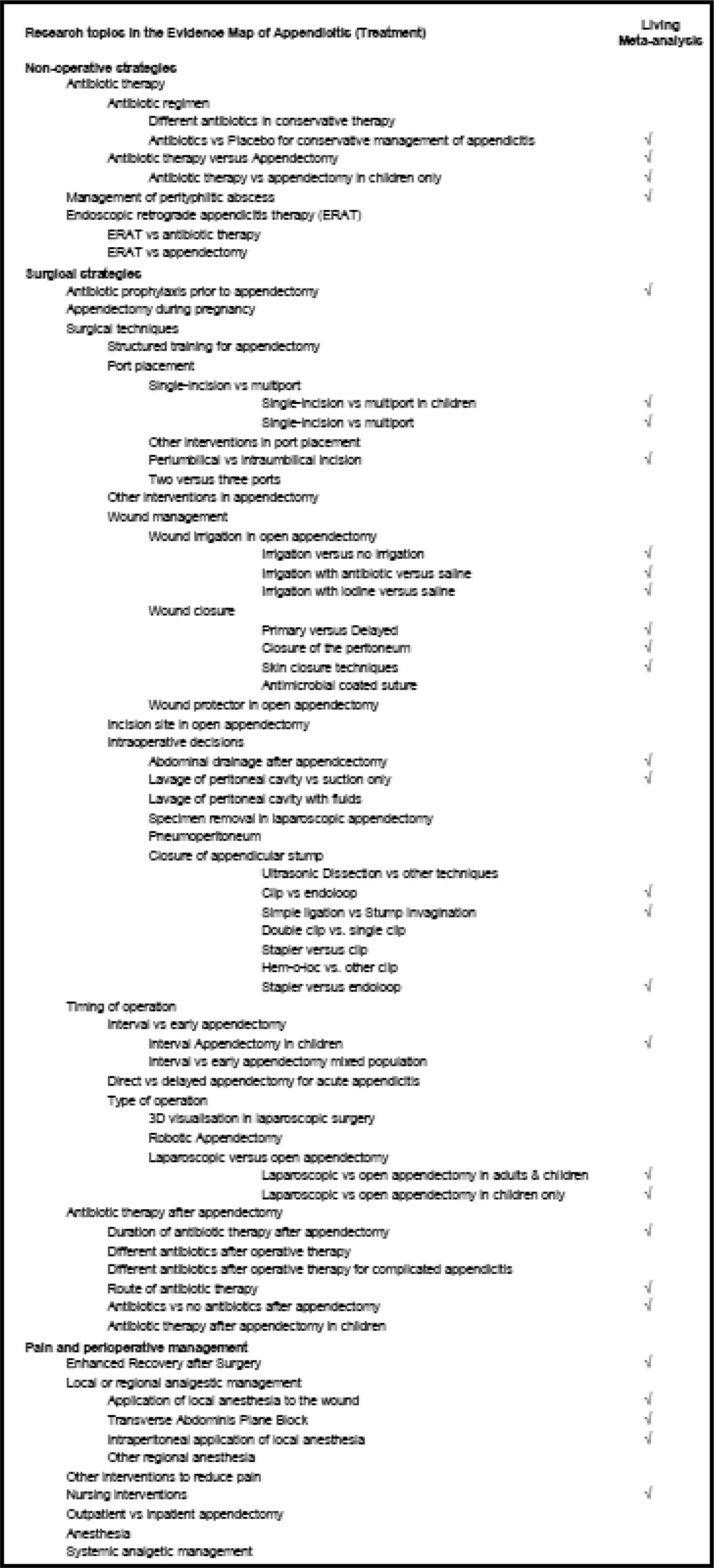
Tabular view of the research topics of the evidence map of appendicitis

### Critical appraisal (bias)

The overall risk of bias judgment was deemed high in 285 of 527 RCTs (54%). In 184 of 527 RCTs (35%), there were some concerns, and the remaining 58 of 527 RCTs (11%) had an overall low risk of bias. Most of the high risk of bias judgement arose from bias due to deviations from intended intervention (7%) and bias in measurement of outcome (5%) or bias due to randomisation (5%). In Table 2 a detailed overview on all bias domains with judgement is given.

**Table 2:** Risk of bias assessment of included RCTs

Risk	Bias due to randomisation	Bias due to deviations from intended intervention	Bias due to missing outcome data	Bias in measurement of outcome	Bias in selection of the reported result	Overall
Low risk	213 (40%)	176(33%)	451 (86%)	151 (29%)	390 (74%)	58 (11%)
Some concerns	292 (55%)	317 (60%)	68 (13%)	346 (66%)	135 (26%)	184 (35%)
High risk	22 (5%)	34 (7%)	8 (1%)	30 (5%)	2 (0%)	285 (54%)

Patients were blinded in 120 of 527 RCTs (23%) and not blinded in 172 of 527 RCTs (33%). Outcome assessors were blinded in 140 of 527 RCTs (27%) and not blinded in 53 of 527 RCTs (10%). Generally, blinding was unreported in the most RCTs and a detailed analysis for all study contributors is given in Table 3.

**Table 3:** Blinding of study contributors of included RCTs

Study contributor	Blinded	Not blinded	Not reported
Patients	120 (23%)	172 (33%)	235 (44%)
Physicians	83 (16%)	249 (47%)	195 (37%)
Data collectors	123 (23%)	56 (11%)	348 (66%)
Outcome assessors	140 (27%)	53 (10%)	334 (63%)
Statisticians	37 (7%)	9 (2%)	481 (91%)

Funding remained unclear in 390 of 527 RCTs (74%). For those funding was reported, 7% of RCTs (38 of 527) were sponsored by industry and 19% were non-industry funded RCTs (99 of 527).

### Benchmarks

In 71860 patients from 368 studies the occurrence rate of surgical site infections in all patients was 7.9% (95%-CI: 7.2% to 8.7%; range: 0.3% to 99.4%).

The surgical site infection rate in uncomplicated appendicitis was 5.4% (52 studies: 8164 patients; 95%-CI: 4.2% to 6.9%; range: 0.3% to 38%).

After laparoscopic appendectomy surgical site infection occurred in 5.8%, (65 studies: 10818 patients; 95%-CI: 4.7% to 7.3%; range: 0.3% to 37.2%;) compared to surgical site infection after open appendectomy of 10.6% (59 studies: 9879 patients; 95%-CI: 8.3% to 13.3%; range: 0.5% to 99.4%).

The re-admission rate after appendectomy was 3.5% (52 studies: 9284 patients; 95%-CI: 2.6% to 4.7%; range: 0.3% to 15%). The re-operation rate after appendectomy was 1.5% (63 studies: 12147 patients; 95%-CI: 1.1% to 2.1%; range: 0.1% to 17.4%). The re-intervention rate after appendectomy was 1.9% (47 studies: 11720 patients; 95%-CI: 1.3% to 2.8%; range: 0.1% to 19.8%).

## Discussion

The Evidence Map of Appendicitis is a systematic review of literature of all existing evidence from published RCTs, ongoing RCTs and SRs dealing with appendicitis. All relevant studies were grouped in research topics and data from RCTs were pooled in meta-analyses. Consequently, an objective and structured overview on diagnostic and treatment of appendicitis was created. By constantly updating, the Evidence Map of Appendicitis becomes a living and always up-to-date source of information for all physicians managing this common global disease.

Today in the era of artificial intelligence with rapidly increasing AI containing tools, offering clinicians and researchers to collect the existing literature, but despite of this the platform of EVIglance with its transparency, reproducibility and high quality control standards including the crucially demanding human critical appraisal empowers the user to reach high value data.

Appendicitis is a common and mostly uncomplicated disease. However, there is a small subset of complicated cases with peritonitis or abscess formation with the potential to be life-threatening or at risk for long-term disability. Therefore, appendicitis has a high global burden of disease [22, 23]. Not due to the frequency of severe cases or the complexity of treatment, but because of its prevalence [24].

Despite global mortality rates have decreased since 1990 [25], appendicitis imposes significant costs on healthcare systems and substantial productivity losses for society [26].

The rising complexity to the decision-making process, emphasizes the importance of tailoring treatment to individual patient needs and regional resources [27–32].

The aim of this project was to address this challenge of ongoing discussions in current literature, combined with the high pace of newly published articles by creating the Evidence Map of Appendicitis: a systematic overview of literature that has been designed to be up-to-date, comprehensive, critically appraised, and presented in a lucid and accessible format. For appendicitis, no comparable project has been undertaken. As first of its kind, the Evidence Map of Pancreatic Surgery, was published in 2021 by the International Study Group of Pancreatic Surgery (ISGPS) [33]. Based on this, the Evidence Map of Appendicitis has been configured using the same methodology [34].

Two existing concepts of evidence-based medicine were combined: the living systematic review and evidence mapping. Living reviews address the risk of outdated or redundant data by ensuring continuously updated and valid information [35–37], while evidence mapping organizes findings into a matrix or map format [38, 39].

The Evidence Map of Appendicitis aims to benefit physicians, researchers, patients, and funding bodies alike. Physicians gain rapid access to current treatment options and their comparative effectiveness, enabling evidence-based decisions tailored to individual patients and clinical situations. With EVIglance, a physician is able to find specific literature to a clinical question five times faster compared to a standard search in Pubmed [40]. Moreover, an overview in high-quality articles is particularly crucial in developing countries, where access to primary research articles is limited. Researchers can identify evidence gaps, focus on novel questions, and avoid redundant studies, while funding bodies are empowered to prioritize impactful projects that address critical evidence gaps. Patients and students benefit from clear and accessible information for education and decision-making. Looking ahead, further evidence maps for other medical topics are planned by the EVIglance initiative (www.EVIglance.com).

### Limitations

While the Evidence Map of Appendicitis provides a comprehensive and up-to-date resource, certain limitations should be acknowledged. While the Evidence Map approach offers a broad overview of available evidence, it cannot replace a well-conducted systematic review that deeply explores a specific research question or topic.

The strength of the evidence map enables rapid navigation through the current literature and provides primary study data. However, it would be in the scope of a single systematic review to provide an in-depth interpretation of this data, which is beyond the scope of evidence mapping.

A single aggregated bias rating per research topic may not fully capture outcome-specific variations in bias. This represents a limitation inherent to the evidence-mapping approach.

## Conclusion

The Evidence Map of Appendicitis is the ultimate go-to source for evidence on appendicitis. It is constantly updated and freely accessible via www.EVIglance.com and as a mobile phone app. Clinical decision-making and evidence-based patient information are supported by the primary data provided, as well as by living meta-analyses.

## Supplementary Information

Below is the link to the electronic supplementary material.

Supplementary file1 (DOCX 14 KB)

Supplementary file2 (DOCX 26 KB)

Supplementary file3 (PDF 468 KB)

## Data Availability

Data is accesible at: www.EVIglance.com
