# Supplementary material for: Evidence map of appendicitis – a living systematic review with meta-analyses
Source: Langenbecks Arch Surg. 2026 Jul 1;411(1):243. doi: 10.1007/s00423-026-04113-3 (PMC13593621; doi:10.1007/s00423-026-04113-3)
Supplement: Supplementary file 1 [file 423_2026_4113_MOESM3_ESM.pdf]

EVI glance

Evidence Map of Appendicitis

Table of contents

Citation and Abbreviations

Risk of Bias summary

Bubble plot

- Operation vs Intervention
- Disease vs Intervention
- Disease vs Operation

Benchmarks

- SSI after appendectomy in all appendicitis
- SSI after appendectomy in uncomplicated appendicitis
- SSI after laparoscopic appendectomy in all appendicitis
- SSI after open appendectomy in all appendicitis
- Re-Admission after appendectomy
- Re-Operation after appendectomy
- Re-Intervention after appendectomy

Please cite as: **Kleindienst et al. EVIglance (online only). 2025.**

Version date: **June 13, 2026**

Abbreviations

CCI: Comprehensive Complication Index; SSI: Surgical Site Infection

Regions

Europe	North America	South America	Africa	Asia	Australia/New Zealand	Total

LEXINGTON MEDICAL

Platin Sponsor

	N	%	N	%	N	%	N	%	N	%	N	%	
RCT	176	32	84	15	6	1	24	4	237	43	20	4	547
SR	176	38	73	16	18	4	14	3	165	35	19	4	466
Protocol	18	27	8	12	1	1	5	7	34	51	1	1	67

Risk of Bias summary

	Randomisation	Deviations	Missing data	Measurement	Selection	Overall
**Low risk**	213 (40%)	176 (33%)	451 (86%)	151 (29%)	390 (74%)	58 (11%)
**Some concerns**	292 (55%)	317 (60%)	68 (13%)	346 (66%)	135 (26%)	184 (35%)
**High risk**	22 (5%)	34 (7%)	8 (1%)	30 (5%)	2 (0%)	285 (54%)

	Patients	Physicians	Data collectors	Outcome assessors	Statisticians
**Blinded**	120 (23%)	83 (16%)	123 (23%)	140 (27%)	37 (7%)
**Not blinded**	172 (33%)	249 (47%)	56 (11%)	53 (10%)	9 (2%)
**Not reported**	235 (44%)	195 (37%)	348 (66%)	334 (63%)	481 (91%)

	Funding source
**Industrial**	38 (7%)
**Non-industrial**	99 (19%)
**Unclear**	390 (74%)

Risk of Bias

Randomized Controlled Trials

	Primary Endpoint	Randomisation[1]	Deviations[2]	Missing data[3]	Measurement[4]	Selection[5]	Overall[6]
Athar 2022		🟡	🔴	🔴	🟢	🟢	🔴
Vejdan 2021	Duration of operation	🟡	🟡	🟢	🟡	🟢	🔴
Hamill 2017	Pain	🟢	🟢	🟢	🟢	🟢	🟢
Kang 2018	Other	🟢	🟢	🟢	🟡	🟢	🟡
Ergin 2022	Pain	🟡	🟡	🟢	🟡	🟢	🔴
Edwards 2011	Pain	🟡	🟢	🟢	🟢	🟢	🟡
Jan 2021	safety/ effectiveness	🟡	🟡	🟢	🟡	🟡	🔴
Naz 2023	effects of menthol gum chewing on nausea, vomiting, and length of hospital stay after appendectomy in children.	🟡	🟡	🟢	🟡	🟢	🔴
Li 2024	study the application value of ERAS	🟡	🟢	🟢	🟡	🟢	🟡
Lan 2024	Other	🟡	🟢	🟢	🟢	🟡	🟡

LEXINGTON MEDICAL
Platin Sponsor

St Peter 2011	Surgical site infect	🟢	🟡	🟢	🟡	🟢	🟡
Ignacio 2004	Pain	🟢	🟢	🟢	🟢	🟡	🟡
Irpe 2024	Pain	🔴	🟡	🟢	🔴	🟡	🔴
Kang 2010	Pain	🟢	🟢	🟢	🟢	🟡	🟡
Hall 2017	Other	🟢	🟡	🟢	🟡	🟡	🔴
Muñoz-Cruzado 2024	Other	🟢	🟡	🟢	🟡	🟢	🟡
Refaat 2015	Pain	🟡	🟡	🟢	🟡	🟢	🔴
Kaplan 2009	Quality of life	🟢	🟡	🟢	🟡	🟡	🔴
Robert 2007	Return to work	🟡	🟢	🟢	🟢	🟢	🟡
Yellin 1993	clinical efficacy of the two regimens	🟡	🟡	🟢	🟡	🟢	🔴
Kim 2011	Pain	🟢	🟢	🟢	🟢	🟢	🟢
Elnabtity 2018	Pain	🟢	🟢	🟢	🟢	🟢	🟢
Edwards 2004	Pain	🟢	🟢	🟢	🟢	🟢	🟢
Jalava 2023	Other	🟢	🟡	🟢	🟢	🟡	🟡
Saha 2010	Surgical site infect	🟢	🟡	🟢	🟡	🟡	🔴
St Peter 2008	Length of hospital stay	🟢	🟡	🟢	🟡	🟢	🟡
Lee 2018	effect of lidocaine patch on postoperative pain control in laparoscopic appendectomy	🟡	🟢	🟢	🟢	🟢	🟡
Lohsiriwat 2005	he efficacy of pre-incisional analgesic bupivacaine infiltration (preemptive analgesia) on postoperative pain relief after appendectomy.	🟡	🟢	🟢	🟢	🟢	🟡
Nunez-Venzor 2021	Length of hospital stay	🟡	🔴	🟢	🟡	🟡	🔴
Javadi 2017	safety and feasibility of open appendectomy usinf small access incision	🟡	🟡	🟢	🟡	🟢	🔴
Ahmed 2020	The objective was to compare the outcomes in cases with early vs delayed oral feeding after appendice ctomy.	🟡	🟡	🟢	🟡	🟢	🔴
Zhang 2023	The degree of pain, recovery time of intestinal function, length of hospital stay, hospital costs, postoperative complications and parental satisfaction	🟡	🟡	🟢	🟡	🟢	🔴
Tarar 2023		🟡	🟡	🟢	🟡	🟢	🔴
Noori 2023	Other	🟡	🟡	🟢	🟡	🟢	🔴
Ergin 2022	Pain	🟡	🟢	🟢	🟢	🟢	🟡

Platin Sponsor

Selänne 2024	Other	🟢	🟡	🟢	🟡	🟢	🟡
Trejo-Ávila 2019	Length of hospital stay	🟡	🟡	🟢	🟡	🟢	🔴
Kocataş 2013	Other	🟡	🟡	🟢	🟡	🟢	🔴
Tlacuilo-Parra 2018	The efficacy and safety measures were length of hospital stay and proportion of complications.	🟢	🟡	🟢	🟡	🟡	🔴
Karadayi 2003	postoperative acute-phase response	🟢	🟡	🟢	🟡	🟢	🟡
Perez 2013	operative time comparing patients undergoing SILS (SILS-A) with those undergoing conventional laparoscopic (LAP-A) appendectomy	🟢	🟡	🟢	🟡	🟢	🟡
Baird 2019	Other	🟢	🟢	🟢	🟢	🟢	🟢
Sadraei-Moosavi 2017	Surgical site infect	🟡	🟡	🟢	🟡	🟢	🔴
Randall 2010	Pain	🟡	🟢	🟢	🟢	🟡	🟡
Saar 2019	To investigate noninferiority of a short and fixed (24 hours) antibiotic administration compared to extended treatment after source control in complicated appendicitis. Primary outcomes included composite postoperative complications and Comprehensive Complication Index (CCI)	🟡	🟡	🟢	🟡	🟡	🔴
Chen 2017		🟡	🟡	🟢	🟡	🟡	🔴
Nechay 2020	Length of hospital stay	🟡	🟡	🟢	🟡	🟢	🔴
Duza 2019	The aim of this work was to compare the results of CLA versus SPLA in patients diagnosed with Acute Appendicitis.	🟢	🟡	🟢	🟡	🟢	🟡
James 2020	Other	🟡	🟢	🟢	🟢	🟢	🟡
Mennie 2020	Surgical site infect	🟡	🟢	🟢	🟡	🟢	🟡
Sippola 2021	Other	🟡	🟢	🟢	🟡	🟢	🟡
Talan 2017	Other	🟡	🟡	🟢	🟢	🟢	🟡
Kuzma 2008	Length of hospital stay	🟡	🟡	🟢	🟡	🟢	🔴
Ali 2018	Surgical site infect	🟡	🟢	🟢	🟡	🟢	🟡

Frutos 2013	Other	🟢	🟡	🟢	🟡	🟢	🟡
Mamidala 2024	Pain	🟢	🟢	🟢	🟢	🟢	🟢
Wright 1993	Pain	🟢	🟢	🟢	🟢	🟢	🟢
Elwahab 2025	Other	🟢	🟢	🟢	🟢	🟡	🟡
Kang 2021	Other	🟢	🟡	🟢	🟡	🟢	🟡
Patkova 2020	Re-Intervention	🟡	🟡	🟢	🟢	🟢	🟡
Ying 2025		🟡	🟢	🟢	🟡	🟡	🔴
McGreal 2002	Surgical site infect	🟢	🟡	🟢	🟡	🟡	🔴
Chiam 1983	effect of metronidazole and cotrimoxazole on the incidence of wound infection following appen dicectomy	🟡	🟡	🟡	🟡	🟡	🔴
Turhan 2009	safety and cost effectiveness	🟡	🟡	🟢	🟡	🟢	🔴
Dass 1989	Surgical site infect	🟡	🟡	🟢	🟡	🟡	🔴
Hopkins 1994	Surgical site infect	🟢	🟢	🟢	🟢	🟢	🟢
Berne 1993	Length of hospital stay	🟢	🟢	🟢	🟢	🟡	🟡
Bennion 1992	efficacy of 2 single-agent drug	🟡	🟡	🔴	🟡	🟡	🔴
Khan 2024	Other	🟢	🟡	🟢	🟡	🟢	🟡
Katkhouda 2006	Morbidity (CCI)	🟢	🟢	🟢	🟢	🟢	🟢
Long 2001	Length of hospital stay	🟢	🟡	🟢	🟡	🟢	🟡
Pedersen 2001	Length of hospital stay	🟢	🟡	🟢	🟡	🟢	🟡
Bauwens 1998	duration of work disability	🟡	🟡	🟢	🟡	🟢	🔴
Heikkinen 1998	Costs	🟢	🟡	🟢	🟡	🟢	🟡
Macarulla 1997	Surgical site infect	🟡	🟡	🟢	🟡	🟡	🔴
Lavonius 2001	operative findings, operative time (from skin incision to skin closure), postoperative complications and the length of hospital stay.	🟢	🟡	🟢	🟡	🟢	🟡
Perez Otero 2022	1-year success rate of antibiotics-alone and quality-of-life measures	🟡	🟡	🟡	🟡	🟢	🔴
Aday 2024	Compare the reliability of a single hem-o-lok clips with a double hem-o-lok clips	🟡	🟡	🟢	🟡	🟢	🔴
Rajkhowa 2016	Wound complication rate, LOS, tramadol-consumption at 1. POD	🟡	🟡	🟢	🟢	🟢	🟡

LEXINGTON MEDICAL
Platin Sponsor

Pettigrew 1981	Surgical site infect	🟢	🟡	🟢	🟡	🟢	🟡
Quddus 2023	Surgical site infect	🔴	🟡	🟢	🟡	🟢	🔴
Salo 1981	Surgical site infect	🟢	🟢	🟢	🟡	🟡	🟡
Donovan 1979	Surgical site infect	🟢	🟢	🟡	🟢	🟡	🟡
Foster 1978	Surgical site infect	🟢	🟢	🟢	🟡	🟡	🟡
Park 2017	Events in antibiotic therapy	🟡	🟡	🟢	🟡	🟢	🔴
Fatima-Tu-Zahara 2020	incidence of surgical site infections and postoperative pain	🟡	🟡	🟢	🟡	🟡	🔴
Tanphiphat 1978	Surgical site infect	🟡	🔴	🟢	🟡	🟡	🔴
Kim 2023	Other	🟡	🟡	🟢	🟡	🟢	🔴
Ghimire 2017	Pain	🟢	🟢	🟢	🟢	🟡	🟡
Shirdashtzadeh 2011	Other	🟢	🟢	🟢	🟢	🟢	🟢
Morton 1999	Other	🟢	🟢	🟢	🟡	🟡	🟡
Sims 1994	Pain	🟢	🟢	🟢	🟢	🟢	🟢
Porteous 1985	Surgical site infect	🟡	🔴	🟢	🔴	🟡	🔴
Gómez-Alonso 1984	Surgical site infect	🟢	🟡	🟢	🟡	🟢	🟡
Hutchinson 1983	Surgical site infect	🟢	🟢	🟢	🟡	🟢	🟡
Viitanen 1984	Surgical site infect	🔴	🟡	🟢	🟡	🟢	🔴
McLean 1983	Surgical site infect	🟡	🔴	🟢	🟡	🟡	🔴
Gledhill 1983	Surgical site infect	🔴	🟡	🟢	🟡	🟢	🔴
Busuttil 1981	Surgical site infect	🟢	🟢	🟢	🟢	🟢	🟢
Bates 1980	Surgical site infect	🟢	🟡	🟢	🟡	🟢	🟡
Morris 1980	Surgical site infect	🟢	🟢	🟢	🟢	🟢	🟢
Taguchi 2016	Other	🟢	🔴	🟢	🟡	🟡	🔴
Lau 1986	Surgical site infect	🟢	🟡	🟢	🟢	🟢	🟡
Greenall 1979	Surgical site infect	🟢	🟢	🟢	🟢	🟡	🟡
Iqbal 2019	Wound Infection And Hospital Stay Between Primary Closure And Delayed Primary Closure In Patients With Perforated Appendicitis	🟡	🟡	🟢	🟡	🟢	🔴
Everson 1977	Surgical site infect	🟡	🟡	🟡	🟡	🟢	🔴
Magarey 1971	Other	🟡	🟡	🟡	🟡	🟡	🔴
Lee 2023	Duration of operation	🟡	🟡	🟢	🟢	🟡	🔴
Soudagar 2024	Effectiveness pain relief	🟡	🟢	🟢	🟢	🟡	🟡
Delvadiya 2024	hemodynamic stability in	🟡	🟢	🟢	🟢	🟡	🟡

LEXINGTON MEDICAL
Platin Sponsor

	laproscopic appendicetomy.						
Ahmed 1987	Surgical site infect	🟢	🟢	🟢	🟡	🟢	🟡
Dash 2024	Other	🟢	🟢	🟢	🟢	🟢	🟢
Wolfe 2004	Pain	🟢	🟢	🟡	🟢	🟢	🟡
Doyle 1994	Pain	🟢	🟡	🟢	🟡	🟢	🟡
Doyle 1994	Pain	🟢	🟢	🟢	🟢	🟢	🟢
Wetter 1991	Surgical site infect	🟢	🟢	🟢	🟢	🟢	🟢
Bauer 1989	Surgical site infect	🟢	🟢	🟢	🟢	🟢	🟢
Onwuanyi 1990	Surgical site infect	🔴	🟡	🟢	🔴	🟡	🔴
Amgwerd 1981	Surgical site infect	🟡	🔴	🟢	🟡	🟡	🔴
Thomson 1987	Surgical site infect	🟡	🔴	🟢	🔴	🟡	🔴
Salem 1979	Surgical site infect	🟢	🟡	🟡	🟡	🟡	🔴
Klima 1998		🟡	🟡	🟡	🟡	🟢	🔴
El-Sefi 1986	Surgical site infect	🟢	🟡	🟢	🟡	🟢	🟡
Saario 1981	Surgical site infect	🔴	🔴	🟢	🟡	🟡	🔴
Thomson 2015	Primary outcomes: intra-operative duration, the rate of wound sepsis, the number of re-operations, the length of hospital stay and the rate of re-admissions	🟡	🟡	🟢	🟡	🟢	🔴
Tanner 1980	Surgical site infect	🟡	🟢	🟢	🟢	🟢	🟡
Nyström 1980	Surgical site infect	🟡	🟡	🟡	🟡	🟢	🔴
Khan 2012	surgical wound infection, length of hospital stay and patient convenience	🟡	🟡	🟢	🔴	🟢	🔴
Greenall 1978	Surgical site infect	🟡	🔴	🟢	🟡	🟡	🔴
Dogar 2020	post-operative outcomes of single incision versus conventional three port laparoscopic appendectomy	🟡	🟡	🟢	🟡	🟢	🔴
Pakniyat 2024	Pain	🟢	🟡	🟢	🟡	🟢	🟡
Lang 1988	Surgical site infect	🟢	🟢	🟡	🟢	🟢	🟡
Tsang 1992	Surgical site infect	🟡	🟢	🟢	🟡	🟢	🟡
Scalise 2024	Pain	🟡	🟡	🟢	🟡	🟢	🔴
Rodgers 1979	Surgical site infect	🟢	🟢	🟡	🟡	🟡	🔴
Adib-Hajbaghery 2013	pain, nausea, and vomiting after appendectomy.	🟡	🟡	🟢	🟡	🟡	🔴

LEXINGTON MEDICAL
Platin Sponsor

Khoori 2024	Pain	🟢	🟢	🟢	🟢	🟢	🟢
Khoramvar 2024	Pain	🟡	🟢	🟢	🟢	🟢	🟡
Dix 2003	Other	🟢	🟢	🟢	🟢	🟢	🟢
Eriksson 1995	Other	🟢	🟡	🟢	🟢	🟢	🟡
Eriksson 1995	Pain	🟢	🟡	🟢	🟡	🟢	🟡
Brennan 1982	Surgical site infect	🟡	🔴	🟢	🟡	🟡	🔴
Salam 1994	Surgical site infect	🟢	🟢	🟢	🟡	🟢	🟡
Kizilcan 1992	Surgical site infect	🟡	🟡	🟢	🟡	🟢	🔴
Siribumrungwong 2018	Surgical site infect	🟢	🟡	🟢	🟢	🟢	🟡
Birkigt 1991	Surgical site infect	🟡	🟡	🟢	🟡	🟡	🔴
El-Mufti 1989	Surgical site infect	🟡	🟡	🟢	🟡	🟡	🔴
Barth 1988	Surgical site infect	🟡	🔴	🟢	🟡	🟢	🔴
Lau 1981	Surgical site infect	🟢	🟢	🟢	🟢	🟢	🟢
Lv 2016	1) Post-operative complications including IAA, superficial wound infection, appendicular stump leakage, and post□□operative abdominal pain, which was defined as abdominal complaints after surgery requiring prolonged clinical observation or additional biochemical or radiological tests; 2) Re-interventions including percutaneous and/ or transrectal drainage, re-operation (laparoscopy/ laparotomy), and prolonged use of intravenous antibiotics (> 3–5 days); 3) Duration of the operation (the time from skin incision to skin closure), duration of hospital stay, and readmission (the duration of a readmission was included inthe hospital stay calculation)	🟡	🟡	🟡	🟡	🟢	🔴
Chiang 2012	Surgical site infect	🟡	🟡	🟢	🟡	🟢	🔴
Kumar 2004	Morbidity (Clavien-Dindo)	🟡	🔴	🟢	🔴	🟡	🔴

LEXINGTON MEDICAL
Platin Sponsor

Berne 1982	Surgical site infect	🟢	🟢	🟢	🟢	🟢	🟢
Razzaq 2019	Duration of operation	🟡	🟡	🟢	🟡	🟢	🔴
Singh 2017		🟡	🟡	🟢	🟡	🟢	🔴
Mousavi 2002	Pain	🟢	🟢	🟢	🟢	🟢	🟢
Gray 1983	Surgical site infect	🟢	🟡	🟢	🟡	🟡	🔴
Jahan 2023	management of post-appendectomy pain	🟡	🟡	🟢	🟡	🟢	🔴
Ortega 1995	Pain	🟢	🟢	🟢	🟢	🟡	🟡
Negm 2024	postoperative incidence of fecal fistula	🟡	🟡	🟢	🔴	🟡	🔴
Zerem 2007	Other	🟢	🟢	🟢	🟡	🟢	🟡
Drumm 1985	Surgical site infect	🟢	🟢	🟢	🟢	🟡	🟡
Chant 1984	Surgical site infect	🟡	🟢	🟢	🟢	🟢	🟡
St Peter 2012	Other	🟢	🟡	🟢	🟡	🟢	🟡
Sazhin 2020	Other	🟡	🔴	🟡	🔴	🟢	🔴
Alamdari 2018	Pain	🟡	🟡	🟢	🟡	🟢	🔴
Sadat-Safavi 2015	Operative time	🟡	🟡	🟢	🟡	🟢	🔴
Khan 2016	Other	🟡	🟡	🟢	🟡	🟢	🔴
Khan 2021	Operative time, wound infection, wound dehiscence, hospital stay	🟡	🟡	🟢	🟡	🟢	🔴
Verma 2024		🟡	🟡	🟢	🔴	🟡	🔴
Lau 2005	Length of hospital stay	🟢	🟡	🟢	🟢	🟢	🟡
Shubing 1997	Surgical site infect	🟡	🟡	🟢	🟡	🟢	🔴
Ciftci 1997	Surgical site infect	🟢	🟢	🟢	🟡	🟢	🟡
St Peter 2025	Re-Admission	🟢	🔴	🟡	🔴	🟢	🔴
Rojanapirom 1992	Surgical site infect	🟡	🟡	🟢	🟡	🟢	🔴
Engström 1985	Surgical site infect	🟡	🟢	🟢	🟢	🟢	🟡
Yellin 1985	Surgical site infect	🟢	🟢	🟢	🟢	🟢	🟢
Kumar 1997	best option for appendical mass	🟡	🟡	🟢	🟢	🟡	🔴
Ahmad 2021	To compare the hospital stay of patients of appendicitis with conservative or surgical management in context of age and gender.	🟡	🟡	🟢	🟡	🟢	🔴
Gundogan 2023	localization with the optimal usage area.	🟡	🟡	🟢	🟡	🟢	🔴
Khan 2020	hospital stay, complications such as wound infection, perforation,	🟡	🟡	🟢	🟡	🟡	🔴

Platin Sponsor

	nausea vomiting, and abdominal abscess. Recurrence rateafter 1 year						
Mingmalairak 2009	Surgical site infect	🟢	🟢	🟢	🟡	🟡	🟡
Puente 1992	Other	🟡	🔴	🟢	🔴	🟡	🔴
Lau 1985	Surgical site infect	🟢	🟢	🟢	🟢	🟢	🟢
Berne 1987	Surgical site infect	🟢	🟢	🟢	🟢	🟢	🟢
Lau 1986	Surgical site infect	🟢	🟡	🟢	🟢	🟡	🟡
The SCARLESS Study Group 2013	Pain	🟡	🟡	🟡	🟡	🟢	🔴
Ihnát 2021	Te operative time, intraoperative and postoperative complications were the primary outcome measures of the study,	🟡	🟡	🟢	🟡	🟢	🔴
Pinto 1980	Surgical site infect	🟡	🟡	🟡	🟡	🟢	🔴
Javadi 2018		🟡	🟡	🟢	🟡	🟢	🔴
Kakinohana 2000	Pain	🟡	🟡	🟢	🟡	🟢	🔴
Gorecki 2001	Surgical site infect	🟢	🟢	🟢	🟢	🟢	🟢
Winslow 1983	Surgical site infect	🟡	🟢	🟡	🟡	🟢	🔴
Patro 2024	Surgical site infect	🟢	🟡	🟢	🟡	🟢	🟡
Soottiporn 1998	Risk of wound infection	🟡	🟡	🟡	🟡	🟢	🔴
Dai 2023	Surgical site infect	🟢	🟢	🟢	🟡	🟢	🟡
Rihan 2023	Pain	🟢	🟡	🟢	🟢	🟢	🟡
Munawar 2023	outcome of hemostasis	🟡	🟡	🟢	🟡	🟢	🔴
Taylor 2004	Surgical site infect	🟢	🟢	🟢	🟢	🟢	🟢
Sanz Villa 1997	Surgical site infect	🟡	🟡	🟢	🟡	🟢	🔴
Lavonius 1996	Surgical site infect	🔴	🟡	🟢	🟡	🟢	🔴
Ahmed 2015	Pain	🟢	🟢	🟡	🟢	🟢	🟡
Al-Shehri 1994	Surgical site infect	🟢	🟢	🟢	🟡	🟢	🟡
El-Sefi 1989	Surgical site infect	🟡	🟡	🟢	🟢	🟡	🔴
Fan 1988	Surgical site infect	🟡	🟢	🟢	🟢	🟢	🟡
Teoh 2012	Pain	🟡	🟢	🟢	🟢	🟢	🟡
Buckels 1985	the efficacy of cefotetan with that of metronidazole in patients undergoing appendectomy	🟡	🔴	🟡	🟡	🟡	🔴
Kaszyński 2021	Other	🟢	🟡	🟢	🟢	🟢	🟡
Hansson 2009	Other	🔴	🔴	🟡	🔴	🟡	🔴

Platin Sponsor

Sole 1982	Surgical site infect	🟢	🟡	🟡	🟡	🟢	🔴
Gottrup 1980	Assess prophylactic effect of metronidazole on postoperative infection	🟡	🟡	🟡	🟡	🟡	🔴
Suresh 2012	Pain	🟢	🟡	🟢	🟢	🟢	🟡
Muddasar 2017	Pain	🟡	🟡	🟢	🔴	🟡	🔴
Seco 1990	Surgical site infect	🟡	🟡	🟡	🟡	🟢	🔴
Kling 1985	Surgical site infect	🟡	🟡	🟢	🟢	🟢	🟡
Lipping 2024	Other	🟡	🟡	🟢	🟡	🟢	🔴
Minneci 2020	1. disability days 2. success rate of nonoperative management	🔴	🟡	🔴	🔴	🟡	🔴
O'Rourke 1984	Other	🟡	🟢	🟢	🟡	🟢	🟡
Keiser 1983	Surgical site infect	🟡	🟡	🟡	🟡	🟢	🔴
Amoli 2008	Pain	🟢	🟢	🟡	🟢	🟢	🟡
Harahsheh 2002	Length of hospital stay	🟡	🔴	🟢	🔴	🟡	🔴
Dandapat 1992	Surgical site infect	🟡	🔴	🟢	🔴	🟡	🔴
Cervantes-Sánchez 2000	Surgical site infect	🟢	🟡	🟢	🟢	🟡	🟡
Grant 1989	Surgical site infect	🟢	🟡	🟢	🟡	🟡	🔴
Tzovaras 2010	Length of hospital stay	🟡	🟡	🟢	🟢	🟢	🟡
Hall 2021	Other	🟡	🟡	🟡	🟡	🟢	🔴
Park 2010	Other	🟢	🟡	🟢	🟡	🟢	🟡
Pearce 2006	Other	🟡	🟡	🟡	🟡	🟢	🔴
Ullah 2021	Frequency of Wound Infection	🟡	🟡	🟢	🟡	🟢	🔴
Badía 1994	Wound infection	🟡	🟡	🟡	🟡	🟢	🔴
Hansen 1996	Duration of operation	🟢	🟡	🟢	🟡	🟢	🟡
Hajinasrollah 2009	Surgical site infect	🟡	🟡	🟢	🟡	🟢	🔴
Cho 2014	operating time, wound complication, and intraperitoneal morbidities, postoperative pain score and cosmetic result score.	🟡	🟡	🟢	🟡	🟢	🔴
Tijerina 2010	Surgical site infect	🟢	🟢	🟢	🟢	🟢	🟢
Morris 1980	Surgical site infect	🟡	🟡	🟢	🟡	🟢	🔴
Kumarakrishnan 1997	Surgical site infect	🟡	🟡	🟢	🟡	🟢	🔴
Kennedy 2023	The present study attempted to evaluate the ease of use and safety of HEM-	🟡	🟡	🟢	🟡	🟢	🔴

Platin Sponsor

	O-LOK polymer clips compared to Roeder's knot in the appendiceal stump closure in patients undergoing laparoscopic appendectomy.						
Cohen 1993	Other	🔴	🟡	🟢	🟡	🟢	🔴
Zeb 2023	Surgical site infect	🟡	🟡	🟢	🟡	🟡	🔴
Tander 2003	Morbidity (CCI)	🟢	🟡	🟢	🟡	🟢	🟡
Huang 2001	Duration of operation	🟢	🟡	🟢	🟡	🟢	🟡
Nordentoft 2000	Other	🟢	🟢	🟢	🟢	🟢	🟢
Ozmen 1999	Morbidity (CCI)	🟡	🟡	🟢	🔴	🔴	🔴
Minné 1997	Duration of operation	🟢	🟡	🟢	🟡	🟡	🔴
Beldi 2004	Surgical site infect	🟢	🟡	🟢	🟡	🟢	🟡
Mutter 1996	Duration of operation	🟡	🔴	🟢	🟡	🟢	🔴
Martin 1995	Duration of operation	🟡	🟡	🟢	🟡	🟢	🔴
Lujan Mompean 1994	Length of hospital stay	🔴	🟡	🟢	🟡	🟢	🔴
Kollias 1994	Surgical site infect	🔴	🟡	🟢	🟡	🟢	🔴
Liu 2020	Other	🟢	🟡	🟢	🟡	🟢	🟡
Frazee 1994	Return to work	🟡	🟢	🟢	🟡	🟢	🟡
Nazemi 2021	Pain	🟡	🟢	🟢	🟢	🟡	🟡
Iqbal 2015	Surgical site infect	🟡	🟡	🟢	🟡	🟢	🔴
Gasior 2014	Other	🟡	🟢	🟡	🟢	🟡	🔴
Lau 1983	Surgical site infect	🟢	🟡	🟢	🟡	🟢	🟡
Liu 2021	Other	🟢	🟢	🟢	🟡	🟢	🟡
Bueno 2005	Postoperative infection rate, length of stay, costs	🟡	🟡	🟢	🟢	🟡	🔴
Colak 2013	clinical outcomes of hem-o-lok ligation system in laparoscopic appendix stump closure by comparing the endoloop ligature	🟢	🟡	🟢	🟡	🟢	🟡
Taylor 2000	determine Presented at the American College of Surgeons, Southern Cali-fornia Chapter Meeting, January, 22, 2000, Huntington Beach, California. Address corres pondence and reprint requests to Edward W. Tay-lor, M.D., F.A.C.S.,	🟡	🟢	🟢	🟡	🟢	🟡

LEXINGTON MEDICAL
Platin Sponsor

	Department of Surgery, Kern Medical Center, 1830 Flower Street, Bakersfield, CA 93305. whether having no minimum IV antibiotic requirement had an effect on morbidity, IV antibiotic use, and hos⍰ pital stay.						
Kye 2013	Duration of operation	🟡	🟡	🟢	🟡	🟢	🔴
Ratnakar 2023	Surgical site infect	🟡	🟡	🟢	🟡	🟢	🔴
Carter 2014	Pain	🟡	🟢	🟡	🟡	🟢	🔴
Akash 2023	Surgical site infect	🟡	🟡	🟢	🟡	🟢	🔴
Sozutek 2013	Pain	🟡	🟡	🟢	🟡	🟢	🔴
Hansson 2009	Other	🔴	🟡	🟢	🟡	🟢	🔴
Schultz 1983	Surgical site infect	🟡	🟡	🟢	🟡	🟢	🔴
Pang 2021	Other	🟢	🟡	🟢	🟢	🟢	🟡
Lee 2009	Surgical site infect	🟡	🟡	🟢	🟢	🟢	🟡
Kum 1993	Surgical site infect	🟢	🟡	🟢	🟡	🟢	🟡
Seco 1985	Other	🟡	🟡	🟡	🟡	🟢	🔴
Meller 1991		🟡	🟡	🟡	🟡	🟡	🔴
Kooi 1990	Surgical site infect	🟡	🟡	🟢	🟡	🟢	🔴
Foster 1981	Surgical site infect	🟡	🟡	🟡	🟡	🟢	🔴
Miholic 1983		🟡	🟡	🟡	🟡	🟢	🔴
Rickett 1969	Surgical site infect	🟡	🟢	🟢	🟡	🟢	🟡
Patel 2024	Other	🟢	🟡	🟢	🟢	🟢	🟡
Tate 1993	Length of hospital stay	🟢	🟢	🟡	🟡	🟢	🟡
Ranjan 2024	Length of hospital stay	🔴	🔴	🟢	🔴	🟡	🔴
Sun 2018	Surgical site infect	🟡	🟡	🟢	🟡	🟢	🔴
Rajabi-Mashhadi 2012	Other	🟡	🟡	🟢	🟡	🟢	🔴
O'Leary 2021	Other	🟢	🟢	🟢	🟢	🟢	🟢
Flum 2020	Quality of life	🟢	🟡	🟢	🟡	🟢	🟡
Schmitt 1989	Other	🟡	🟡	🟢	🟡	🟢	🔴
Almagor 2005	Other	🟢	🟡	🟢	🟢	🟢	🟡
Vons 2011	Other	🟢	🔴	🟢	🟡	🟡	🔴
Svensson 2015	Other	🟡	🟡	🟢	🟡	🟢	🔴
Lejus 1996	Pain	🟢	🟡	🟢	🟡	🟡	🔴
de Wijkerslooth 2023	Other	🟢	🟡	🟢	🟡	🟢	🟡
Youssef 2021	Feasibility, safety and efficacy of ligation method or clip application, operative time,	🟢	🟡	🟢	🟡	🟡	🔴

Platin Sponsor

	postoperative outcome and complications.						
Bostan 2023	Surgical site infect	🟡	🟢	🟢	🟡	🟢	🟡
Gutierrez 1987	Surgical site infect	🟡	🟡	🟢	🟡	🟢	🔴
Corder 1983	Surgical site infect	🟡	🟢	🟢	🟢	🟢	🟡
Baba 2023	safety, effectiveness, length of surgery, complications before and after the procedure, and length of hospital stay	🟡	🟡	🟢	🟡	🟡	🔴
Sellappan 2024	Surgical site infect	🟢	🟢	🟢	🟢	🟢	🟢
Olmi 2005	Duration of operation	🔴	🟡	🟢	🟡	🟢	🔴
Moberg 2005	Other	🟢	🟢	🟢	🟢	🟢	🟢
Eskandarlou 2018	To compare feasibility, safety, and cosmetic outcomes of new right groin incision with a classic McBurney's incision.	🟡	🟡	🟢	🔴	🟡	🔴
Little 2002	Length of hospital stay	🟢	🔴	🟡	🔴	🟡	🔴
Hay 1998	Re-Operation	🔴	🟡	🟢	🟡	🟢	🔴
Blakely 2011	Other	🟢	🟡	🟢	🟡	🟢	🟡
Benlloch 1995	clinical efficacy and cost/ benefit relation	🟡	🟡	🟡	🟡	🟢	🔴
Banani 1999	Length of hospital stay	🟡	🟡	🟢	🟡	🟢	🔴
Jacobs 1992	comparision 2 methods of appendix stump treatment	🟡	🟡	🟢	🟡	🟢	🔴
Chhakarvarty 2024	Postoperative symptoms	🟡	🟡	🟢	🟡	🟢	🔴
Al-Dhohayan 1993	prevention of sepsis	🟡	🟡	🟢	🟡	🟢	🔴
Buanes 1991	Surgical site infect	🟡	🟡	🟢	🟡	🟢	🔴
Osime 1988	opera. ting time, post-operative pyrexia, wound infection, histology of appendix, iengtn 01 hospital stay anunal follow-up complicatidns	🟡	🟡	🟢	🟡	🟢	🔴
Etienne 2023	Pain	🟢	🟢	🟡	🟢	🟢	🟡
Tejaswi 2023	Surgical site infect	🟡	🟡	🟢	🟡	🟡	🔴
Andrade 2016	Surgical site infect	🟢	🟢	🟢	🟢	🟢	🟢
Ghimire 2015	Pain	🟢	🟢	🟢	🟢	🟡	🟡
Ureyen 2020	Aim to evaluate the superiority	🟡	🟡	🟢	🟡	🟢	🔴

LEXINGTON MEDICAL
Platin Sponsor

	of intracorporeal sutures and Hem-o-lok clips about efficiency, reliability and cost.						
Enochsson 2001	Surgical site infect	🟢	🟡	🟡	🔴	🟡	🔴
Cox 1996	Length of hospital stay	🟡	🔴	🟡	🔴	🟢	🔴
Shen 2022	Other	🟢	🟡	🟢	🟡	🟢	🟡
Andersson 2017	Other	🟡	🟡	🟢	🟡	🟢	🔴
Gemici 2020	Other	🟡	🟡	🟢	🟡	🟢	🔴
Arnold 2018	Other	🟡	🟡	🟢	🟡	🟢	🔴
Ravari 2011	To compare infective complications rate after open appendectomy for nonperforated appendicitis receiving either oral or intravenous metronidazole as prophylaxis.	🟡	🟡	🟢	🟡	🟢	🔴
Sardiwalla 2018	Other	🟢	🟡	🟢	🟢	🟢	🟡
Allo 1999	Other	🟡	🟢	🟡	🟢	🟢	🟡
Salminen 2015	Other	🟢	🟡	🟢	🟡	🟢	🟡
Attwood 1992	not defined	🟡	🟢	🟡	🟡	🟡	🔴
Sethy 2022	wound infection rate	🟡	🟡	🟢	🟡	🟢	🔴
Haijanen 2019	Other	🟢	🟡	🟢	🟡	🟢	🟡
Iresjö 2024	Other	🟡	🟡	🟡	🟡	🟢	🔴
Nataraja 2019	Length of hospital stay	🟡	🟡	🟢	🟡	🟢	🔴
Kouhia 2010	long-term outcome	🟡	🟡	🟢	🟡	🟢	🔴
Lee 2021	Other	🟡	🟡	🟢	🟡	🟢	🔴
Sezeur 1997		🟡	🟡	🟡	🟡	🟢	🔴
Tanphiphat 1976	Surgical site infect	🟡	🟡	🟢	🟡	🟢	🔴
Sherman 1976	incidence of postoperative sepsis or other complications	🟢	🟢	🟢	🟡	🟢	🟡
Sippola 2017	Other	🟡	🟢	🟢	🟢	🟡	🟡
Goudar 2011	primary outcome measures of the patients such as operative duration, hospital stay, post-operative pain and post operative complications	🟡	🟡	🟢	🟡	🟢	🔴
Koluh 2010	time duration of operation was measured, the duration of application of hem-o-lok and endo-loop, postoperative	🟡	🟡	🟡	🟡	🟢	🔴

Platin Sponsor

	analgesia, the duration of hospitalization, intra-operative complications, anatomic position of appendix, appendicitis, and postoperative complications.						
Navarra 2002	LOS, operation time, complication intra and psotoperatively, mortality, pain	🟡	🟡	🟢	🟡	🟢	🔴
Salminen 2018	Late (after 1 year) appendicitis recurrence after antibiotic treatment, complications, length of hospital stay, and sick leave.	🟢	🟡	🟢	🟡	🟢	🟡
Hebebrand 1994	Pain	🟡	🟡	🟡	🟡	🟢	🔴
Olsen 1993	Frequency of unnecessary ap pendicectomy in women	🟡	🟡	🟡	🟡	🟢	🔴
Emile 2020	Surgical site infect	🟢	🟡	🟢	🟢	🟢	🟡
Sirinek 1991	Surgical site infect	🟡	🟡	🟡	🟡	🟢	🔴
King 1983	Other	🟢	🟢	🟢	🟢	🟢	🟢
Human 2022	post-operative complications with and without a peritoneal drain.	🟡	🟡	🟢	🟡	🟢	🔴
Snow 2016	Other	🟡	🟡	🟢	🟡	🟢	🔴
Dogar 2020	To compare the outcomes of periumbilical incision versus intraumbilical incision in patients undergoing laparoscopic appendectomy.	🟡	🔴	🔴	🟡	🟢	🔴
Abdalgaliel 2021	We compare between clipping versus intra-corporeal ligation of appendiceal stump regarding safety, timing of operation and difficulty.	🟡	🟢	🟢	🟡	🟢	🟡
Tighe 1981	Surgical site infect	🟡	🟡	🟡	🟡	🟡	🔴
Birkigt 1989	Surgical site infect	🟡	🟡	🟡	🟡	🟢	🔴
Ali 2020	rate of wound infection	🟡	🔴	🔴	🟡	🟢	🔴
Kazemier 1997	Superiority and safety of LA compared to OA	🟢	🟡	🟢	🟡	🟢	🟡
Hart 1996	efficacy of	🟡	🟡	🟢	🟡	🟢	🔴

LEXINGTON MEDICAL
Platin Sponsor

	laparoscopic appendectomy (Number of days in hospital and time to full recovery)						
Sippola 2020	Quality of life and patient satisfaction at 7 year follow up	🟢	🟡	🟢	🟡	🟢	🟡
Pattanshetti 2018	not clearly defined	🟡	🟡	🟢	🟡	🟢	🔴
Miyano 2019		🔴	🟡	🟡	🟡	🟢	🔴
Rashid 2013	Various intraoperative and postoperative parameters like operative time, postoperative pain, duration of ileus, postoperative stay, and time to return to activities of daily life were evaluated	🟡	🟡	🟢	🟡	🟢	🔴
Armughan 2021	wound infection rate	🟡	🟢	🟢	🟡	🟢	🟡
Tepas 2012	Costs	🟡	🔴	🟡	🟡	🟡	🔴
Khan 2014	Surgical site infection	🟡	🟡	🟢	🟡	🟡	🔴
Khan 2019	wound complication of perforated appendicitis	🟡	🔴	🔴	🟡	🟢	🔴
Javed 2018	To compare frequency of wound infection following open versus laparoscopic appendectomy.	🟡	🟢	🟢	🟡	🟢	🟡
Reiertsen 1995	Other	🟡	🟡	🟢	🟡	🟢	🔴
Bolla 2008	Other	🟡	🟡	🟢	🟡	🟢	🔴
Rafique 2017	infection rate	🟡	🔴	🔴	🟢	🟢	🔴
Neves 1988	ion of operation, complications (wound infection, wound dehiscence, abscess formation, intestinal obstruction, fistula and seroma) or hospital stay.	🔴	🟡	🟡	🟡	🟢	🔴
Teerawiwatchai 2018	Other	🟡	🟡	🟢	🟡	🟢	🔴
Ali 2016	mean operation duration of time	🟡	🟡	🟢	🟡	🟡	🔴
Salkaya 2016	Pain	🟡	🟡	🟢	🟡	🟢	🔴
Mustafa 2016	Surgical site infect	🟡	🟡	🟢	🟡	🟢	🔴
Laine 1997	Other	🟡	🟡	🟢	🟡	🟢	🔴
Bickel 2011	Surgical site infect	🟡	🟢	🟢	🟢	🟢	🟡

Platin Sponsor

Patil 2021	to compare the results of application endoclips like Liga Clips and Hem-o-Lok for CAS in LA in cases of Appendicitis without complications.	🟢	🟡	🟢	🟡	🟢	🟡
Nadeem 2016	https://www.sciencedirect.com/science/article/pii/S240585721530022X	🟡	🟡	🟢	🟡	🟢	🔴
Shaikh 2009	Duration of operation	🟢	🟡	🟡	🟡	🟢	🔴
Qaiser 2021	Duration of operation	🟢	🟢	🟢	🟢	🟢	🟢
Roumen 2009	Pain	🟢	🟢	🟢	🟢	🟢	🟢
Ge 2014		🟡	🟡	🟢	🟡	🟢	🔴
Hellberg 1999	Return to work	🟡	🟡	🟢	🟡	🟢	🔴
Bannon 1997	hospital course, morbidity, time until return to work, and reliable economic outcomes based on total charges and estimated total costs.	🟢	🟡	🟢	🟡	🟢	🟡
Askarpour 2012	Comparison of post-operative C-reactive protein	🟡	🟡	🟡	🟡	🟢	🔴
Clarke 2011	Other	🟢	🟢	🟡	🟢	🟡	🟡
Yu 2013	Other	🟢	🟢	🟢	🟢	🟢	🟢
Hesami 2014	Surgical site infect	🟡	🟡	🟢	🟢	🟢	🟡
Nazir 2019		🟡	🟡	🟢	🟡	🟢	🔴
Soreide 1987	postoperative infection rate	🟡	🟡	🟢	🟡	🟢	🔴
Talha 2020	To compare the clinical outcome of laparoscopic and open appendectomy in perforating appendicitis.	🟡	🟡	🟢	🟡	🟢	🔴
Aktimur 2016	oxidative stress in patients who underwent laparoscopic and OA for uncomplicated acute appendicitis by analysing the level of serum markers of oxidative stress	🟢	🟢	🟢	🟢	🟢	🟢
Yin 2025		🟡	🟢	🟡	🔴	🟡	🔴
Mantoğlu 2015	To determine the feasibility of LA especially in terms of cost effectiveness.	🟡	🟡	🟢	🟡	🟢	🔴
Fraser 2010	Other	🟢	🟢	🟢	🟡	🟢	🟡

Platin Sponsor

Khalil 2011	to compare LA and OA in terms of primary outcome measures, such as operative duration, length of hospital stay, and postoperative complications.	🟡	🟡	🟢	🟡	🟢	🔴
Botteri 2021	Duration of operation	🟢	🟡	🟢	🟡	🟢	🟡
Dalgic 2014	Other	🟡	🟢	🟢	🔴	🟢	🔴
Mentula 2015	Length of hospital stay	🟢	🔴	🟢	🟡	🟡	🔴
Jensen 1984	Other	🟡	🟡	🟡	🟡	🟡	🔴
Wayand 1982	Wound infection	🟡	🟡	🟡	🟡	🟢	🔴
Singh 2023	postoperative pain, mean operating time, duration of hospital stay, and post-operative complications	🟡	🟡	🟢	🟡	🟢	🔴
Appa 2023	complications, morbidity and mortality	🟡	🟡	🟢	🟡	🟢	🔴
Saxena 2023	Pain	🟡	🟡	🟢	🟡	🟢	🔴
Kumari 2024	Surgical site infect	🟢	🟡	🟢	🟡	🟡	🔴
Maske 2024	Surgical site infect	🟡	🟡	🟢	🟡	🟢	🔴
Nayak 2023	Surgical site infect	🟡	🟡	🟢	🟢	🟡	🔴
Adenuga 2024	Pain	🟢	🟢	🟢	🟢	🟢	🟢
Adams 2024	Other	🟡	🟡	🟢	🟡	🟢	🔴
Pauniaho 2010	Surgical site infect	🟡	🟢	🟢	🟡	🟢	🟡
Kim 2012	Other	🟢	🟡	🟢	🟡	🟢	🟡
The CODA Collaborative 2021	Quality of life	🟢	🟢	🟡	🔴	🟡	🔴
Kim 2020	sensitivity and specificity	🟢	🟢	🟢	🟡	🟢	🟡
Mán 2014	to compare the reliability of the Alvarado score and clinical judgment and to refine the score to make it easier to use.	🟡	🟡	🟡	🟡	🟢	🔴
Lastunen 2022	Re-Admission	🟢	🟡	🟢	🟡	🟢	🟡
The CODA Collaborative 2020	Quality of life	🟢	🟡	🟢	🟡	🟢	🟡
Negm 2022	postoperative incidence of fecal fistula and, recurrent collection , and quality of life in each group during the 3-months follow-up period	🟢	🟢	🟢	🟢	🟢	🟢
Salminen 2026	Re-Admission	🟢	🟢	🟢	🟢	🟢	🟢

Platin Sponsor

St Peter 2010	time to regular diet, total length of hospitalization, abscess rate, wound infection rate, doses of narcotics, number of health care visits, number of CT scans, and total hospital charges incurred including interventional radiology charges.	🟡	🟡	🟢	🟡	🟢	🔴
Tan 2020	Other	🟢	🟡	🟢	🟡	🟢	🟡
Minneci 2019	1) caregiver decision self-efficacy immediately after the treatment decision using the Decision Self-efficacy Scale18-20; (2) caregiver health care satisfaction at discharge using the PedsQL (Pediatric Quality of Life Inventory) 4.0 Healthcare Satisfaction Generic Module–Parent Report21,22; and (3) disability days of the patient at 1 year.	🟢	🟡	🟢	🟢	🟢	🟡
The LOCAT Group 2017	Other	🟢	🟡	🟢	🟢	🟢	🟡
Anderson 2020	Other	🟢	🟡	🟢	🟢	🟢	🟡
Vermeulen 1999	Pain	🟢	🟢	🟢	🟢	🟢	🟢
Kılınçer 2017	Other	🟡	🟡	🟢	🟡	🟢	🔴
Gonenc 2012	Other	🟡	🟡	🟢	🟡	🟢	🔴
Fabri 2025		🟢	🟢	🟢	🟢	🟡	🟡
Jumaah 2025	Surgical site infect	🟢	🟢	🟢	🟢	🟡	🟡
Liu 2025	Length of hospital stay	🟡	🟢	🟢	🟡	🟡	🔴
Yang 2024	Other	🟡	🟡	🟢	🟡	🟡	🔴
Enochsson 2004	Other	🟢	🟡	🟢	🟢	🟢	🟡
Xue 2025	Surgical site infect	🟢	🟢	🟢	🟡	🟡	🟡
Hershko 2007	Other	🟢	🟡	🟢	🟡	🟢	🟡
Sharma 2023	compare the results of surgical care versus non-operative treatment (NOT) with antibiotics for childhood appendicitis.	🟡	🟡	🟡	🟡	🟢	🔴

LEXINGTON MEDICAL
Platin Sponsor

Hekimoglu 2011	Other	🟡	🟢	🟢	🟢	🟢	🟡
Erten 2024	Pain	🟢	🟡	🟢	🟡	🟢	🟡
Luksaite-Lukste 2024	Other	🟡	🟡	🟢	🟡	🟢	🔴
Caiazzo 2010		🟡	🟡	🟢	🔴	🔴	🔴
Sindhvananda 2005	Pain	🟡	🟡	🟢	🔴	🟡	🔴
Gavela 2012	Evaluate the use of procalcitonin (PCT) and C-reactive protein (CRP) on admission as predictors of the severity of appendicitis in children.	🔴	🟡	🟢	🟡	🟡	🔴
Ozgur 2025	Pain	🟢	🟢	🟢	🟢	🟡	🟡
Lee 2020	Surgical site infect	🔴	🔴	🟢	🟡	🟡	🔴
Namq 2025		🟡	🟢	🟢	🟡	🟡	🔴
Kar 2025		🟡	🟢	🟢	🟡	🟡	🔴
Mathur 2025	Duration of operation	🟢	🟡	🟢	🟢	🟢	🟡
Srikantegowda 2025	Duration of operation	🟡	🟢	🟢	🟡	🟡	🔴
Tekcan 2026	Pain	🟢	🟢	🟢	🟢	🟢	🟢
Akkoyun 2026	Pain	🟡	🟢	🟢	🟡	🟡	🔴
Schneider 2005	Other	🟡	🟢	🟢	🟢	🟢	🟡
Styrud 2006	Morbidity (CCI)	🟢	🟡	🟢	🟡	🟢	🟡
Conn 1986	Pain	🟡	🟢	🟢	🟢	🟡	🟡
Anderson 2009	Other	🟡	🟡	🟡	🟡	🟢	🔴
López 2022	Length of hospital stay	🟢	🟡	🟢	🟡	🟢	🟡
Pata 2021	Quality of life	🟢	🟢	🟡	🟢	🟢	🟡
Jensen 2004	Other	🟢	🟢	🟢	🟢	🟡	🟡
Abdul-Latif 2025	Surgical site infect	🟢	🟢	🟢	🟢	🟡	🟡
He 2025	Length of hospital stay	🟢	🟢	🟢	🟢	🟢	🟢
Carollo 2021	Duration of operation	🟡	🟢	🟢	🟡	🟡	🔴
Mittal 2004	Other	🔴	🟢	🟢	🟢	🟡	🔴
Wang 2019	Surgical site infect	🟡	🟡	🟢	🟡	🟡	🔴
Shah 2008	main out come measured were, time to operation, hospital stay, any adverse out come, negative appendicectomy and delayed operation leading to perforation.	🟡	🟡	🟢	🟡	🟢	🔴
Erginel 2023	Other	🟡	🟡	🟢	🟡	🟢	🔴
Delibegović 2018	Other	🟡	🟢	🟢	🟡	🟡	🔴
Munro 2002	Other	🟢	🟢	🟢	🟢	🟢	🟢
Thanapal 2014	Other	🟡	🟢	🟢	🟡	🟢	🟡
Pan 2013	feasibility, safety, and	🟡	🟡	🟢	🟡	🟢	🔴

Platin Sponsor

	cosmetic results of transumbilical single incision laparoscopic appendectomy (TSILA), using a single working channel with conventional instruments						
Daly 2026	Pain	Green	Green	Green	Green	Green	Green
Shrestha 2025	Surgical site infect	Green	Green	Green	Green	Yellow	Yellow
Abdullah 2025	Duration of operation	Green	Green	Green	Yellow	Yellow	Yellow
Christudhas 2025	Pain	Green	Green	Green	Green	Yellow	Yellow
Kishore 2025	Duration of operation	Yellow	Green	Green	Yellow	Yellow	Red
Bloy 2026	Pain	Green	Green	Green	Green	Green	Green
Nagalekshmi 2025	Pain	Yellow	Green	Green	Red	Yellow	Red
Kleif 2018	Pain	Green	Green	Green	Green	Green	Green
Zhu 2025	Length of hospital stay	Green	Green	Green	Yellow	Green	Yellow
Thakur 2025	Pain	Green	Green	Green	Green	Yellow	Yellow
Tran 2023	Surgical site infect	Yellow	Green	Green	Yellow	Yellow	Red
Mori 2014	Duration of operation	Green	Yellow	Green	Yellow	Green	Yellow
Ahluwalia 2025	Pain	Green	Green	Green	Green	Yellow	Yellow
Shehata 2025	Surgical site infect	Green	Green	Green	Green	Green	Green
Miao 2025	Quality of life	Green	Green	Green	Yellow	Yellow	Yellow
Topuz 2026	Duration of operation	Yellow	Green	Green	Yellow	Green	Yellow
Foster 1987	Surgical site infect	Yellow	Red	Green	Red	Yellow	Red
Bektasoglu 2019	Pain	Green	Green	Green	Green	Green	Green
Salminen 2022	Other	Green	Green	Green	Yellow	Green	Yellow
Mahmood 2021	infection risks, hospital stay, operative time, and cost..	Yellow	Yellow	Green	Yellow	Green	Red
Desai 2015	Other	Yellow	Yellow	Green	Yellow	Green	Red
Chin 1989	Other	Green	Green	Green	Green	Green	Green
Foster 1986	Surgical site infect	Green	Yellow	Green	Green	Green	Yellow
Rosenfeld 2018	Other	Yellow	Yellow	Green	Yellow	Green	Red
Lintula 2001	Other	Yellow	Red	Green	Red	Green	Red
Golebiewski 2019	Other	Yellow	Yellow	Green	Yellow	Green	Red
Larsson 2001	Other	Green	Yellow	Green	Yellow	Green	Yellow
Bartın 2016	Degree of surgical stress in open vs. laparoscopic appendectomy	Yellow	Yellow	Green	Yellow	Green	Red
Liberman 1995	Surgical site infect	Green	Green	Green	Green	Green	Green
Douglas 2000	Length of hospital stay	Green	Yellow	Green	Yellow	Green	Yellow
Sajjad 2021	Other	Yellow	Yellow	Green	Yellow	Green	Red
Feng 2023	Pain	Green	Green	Green	Yellow	Green	Yellow

Platin Sponsor

Park 2021	Pain	🟢	🟡	🟢	🟢	🟢	🟡
Courrèges 1996	Pain	🟢	🟢	🟢	🟢	🟢	🟢
Söderquist-Elinder 1995	Surgical site infect	🟡	🟢	🟢	🟡	🟢	🟡
McAnena 1992	Surgical site infect	🔴	🟡	🟢	🟡	🟢	🔴
Ceresoli 2019	Other	🟡	🟡	🟢	🟡	🟢	🔴
Nechay 2018	Length of hospital stay	🟡	🟡	🟢	🟡	🟢	🔴
Samee 2023	To determine the role of metronidazole after appendice ctomy in simple cases of acute appendicitis	🟡	🟡	🟢	🟡	🟢	🔴
Mahmood 2016	Surgical site infect	🟡	🟡	🟢	🟡	🟢	🔴
Kotaluoto 2012	Surgical site infect	🟡	🟡	🟢	🟡	🟢	🔴
Henle 1996		🟡	🟡	🔴	🟡	🟢	🔴
Willard 1997	Postoperative analgesic requirements, pain assessment by visual analogue scale and length of hospital sta	🟢	🟢	🟢	🟢	🟢	🟢
McIntosh 1984	Surgical site infect	🟡	🟡	🟢	🟢	🟢	🟡
Pokorny 1991		🟡	🟡	🟡	🟡	🟡	🔴
Schropp 1991	efficacy, safety and cost-effectiveness	🟢	🟢	🟢	🟡	🟢	🟡
Kekomäki 1981	Length of hospital stay	🟡	🟡	🟡	🟡	🟢	🔴
Ahmed 2020	Length of hospital stay	🟡	🟢	🟢	🟡	🟡	🔴
Kargar 2011	Morbidity (Clavien-Dindo)	🟢	🟡	🟢	🟡	🟢	🟡
Smania 2008	Other	🟢	🟢	🟢	🟢	🟡	🟡
Malik 2007	Pain	🟢	🟢	🟢	🟢	🟡	🟡
Farooqi 2021	Surgical site infect	🟡	🟡	🟢	🟡	🟢	🔴
Cho 2022	Other	🟡	🟢	🟢	🟡	🟢	🟡
Anderson 2010	Other	🟡	🟡	🟢	🟡	🟡	🔴
Mittal 2004	Other	🟡	🟡	🟢	🟡	🟢	🔴
Kaiser 2002	Other	🟢	🟡	🟢	🟡	🟢	🟡
Aderounmu 2024	Surgical site infect	🟡	🟢	🟢	🟢	🟢	🟡
Kang 2015	Other	🟢	🟢	🟢	🟢	🟢	🟢
Jan 2011	Other	🟡	🟡	🟢	🟡	🟢	🔴
Eldemrdash 2025	Pain	🟢	🟢	🟢	🟢	🟢	🟢
Krishna 2021	Preventing negative laparotomies in acute appendicitis.	🟡	🟡	🟢	🟡	🟡	🔴
Lintula 2009	Other	🟡	🟡	🟢	🟡	🟢	🔴
Hong 2003	Other	🟡	🟡	🟡	🟡	🟢	🔴
Calderon-Alvarado	Length of hospital stay	🟢	🟢	🟢	🟢	🟢	🟢

LEXINGTON MEDICAL
Platin Sponsor

2026							
Chen 2025	Pain	🟢	🟢	🟢	🟡	🟢	🟡
Jalava 2025	Other	🟢	🟢	🟢	🟢	🟢	🟢
Sahoo 2025	Pain	🟢	🟢	🟢	🟢	🟡	🟡

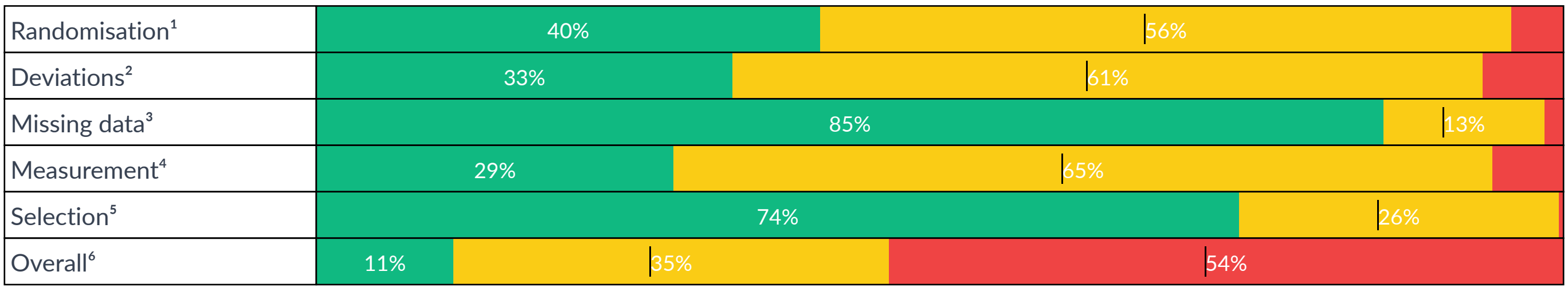

🟢 Low risk 🟡 Some concerns 🔴 High risk

1. Bias arising from the randomization process
2. Bias due to deviations from intended interventions
3. Bias due to missing outcome data
4. Bias in measurement of the outcome
5. Bias in selection of the reported result
6. Overall risk of bias

Operation vs Intervention

	Emergency surgery	Antibiotic treatment	Endoscopy	Elective surgery	Interventional radiology	Other
Laparoscopic appendectomy	28598	9632	0	480	172	2650
Mixed	5891	1533	0	0	0	2057
Open appendectomy	16004	20170	0	253	0	1301
None	513	1748	190	91	50	14482

Disease vs Intervention

	Emergency surgery	Antibiotic treatment	Endoscopy	Elective surgery	Interventional radiology	Other
Acute uncomplicated appendicitis	43426	26370	190	264	0	17105
Acute complicated appendicitis	7242	6653	0	360	0	3385
Chronic appendicitis	106	0	0	140	0	0
Perityphlitic abscess	232	60	0	60	222	0

Disease vs Operation

Platin Sponsor

	Laparoscopic appendectomy	Mixed	Open appendectomy	None
Acute uncomplicated appendicitis	33300	8453	32082	13520
Acute complicated appendicitis	7682	1028	5426	3504
Chronic appendicitis	146	0	100	0
Perityphlitic abscess	404	0	120	50

Benchmarks

SSI after appendectomy in all appendicitis

Surgical site infect: 7.9%, 95%-CI: 7.2% to 8.7%, Range: 0.3% to 99.4% (368 studies, 71860 patients, Single-arm proportional meta-analysis)

SSI after appendectomy in uncomplicated appendicitis

Surgical site infect: 5.4%, 95%-CI: 4.2% to 6.9%, Range: 0.3% to 38.0% (52 studies, 8164 patients, Single-arm proportional meta-analysis)

SSI after laparoscopic appendectomy in all appendicitis

Surgical site infect: 5.8%, 95%-CI: 4.7% to 7.3%, Range: 0.3% to 37.2% (65 studies, 10818 patients, Single-arm proportional meta-analysis)

SSI after open appendectomy in all appendicitis

Surgical site infect: 10.6%, 95%-CI: 8.3% to 13.3%, Range: 0.5% to 99.4% (59 studies, 9879 patients, Single-arm proportional meta-analysis)

Re-Admission after appendectomy

Re-Admission: 3.5%, 95%-CI: 2.6% to 4.7%, Range: 0.3% to 15.0% (52 studies, 9284 patients, Single-arm proportional meta-analysis)

Re-Operation after appendectomy

Re-Operation: 1.5%, 95%-CI: 1.1% to 2.1%, Range: 0.1% to 17.4% (63 studies, 12147 patients, Single-arm proportional meta-analysis)

Re-Intervention after appendectomy

Re-Intervention: 1.9%, 95%-CI: 1.3% to 2.8%, Range: 0.1% to 19.8% (47 studies, 11720 patients, Single-arm proportional meta-

LEXINGTON MEDICAL
Platin Sponsor

analysis)

Please note:
All data displayed with this app are part of a living systematic review.
A living systematic review is a scientific endeavor.
The copyright of the included studies is with the respective publishers or authors and full citation is given for every included study.
If you have any questions or you want to do your own living systematic review, please contact info@EVIglance.com

Disclaimer: The sponsor has no influence on the scientific integrity of this evidence map. He played no rule in planning, conduct, analysis or decision to publish the data.

LEXINGTON MEDICAL

Platin Sponsor
