# Supplementary material for: Evidence map of appendicitis – a living systematic review with meta-analyses
Source: Langenbecks Arch Surg. 2026 Jul 1;411(1):243. doi: 10.1007/s00423-026-04113-3 (PMC13593621; doi:10.1007/s00423-026-04113-3)
Supplement: Supplementary file 2 [file 423_2026_4113_MOESM1_ESM.docx]

Appendix 1: Full search strategy for all databases

**Pubmed**

(appendicitis[tiab] OR "Appendicitis"[Mesh] OR (inflamma*[tiab] AND "appendix"[tiab]) OR appendiceal[tiab])

OR ("Appendectomy"[Mesh] OR appendectom*[tiab] OR appendicectom*[tiab])

AND (random*[tw] OR "Randomized Controlled Trial"[pt] OR RCT[tw] OR "Randomized Controlled Trials as Topic"[Mesh] OR "Controlled Clinical Trial" [pt] OR systematic review [pt] OR meta-analysis [pt] OR review [pt] OR meta-analysis [tw] OR review [tw])

NOT (animals [mh] NOT humans [mh])

**Web of Science**

TS = (appendicitis OR (inflamma* AND appendix) OR appendiceal)

OR TS = (appendectom* OR appendicectom*)

AND TS = (random* OR RCT OR "systematic review" OR "meta-analysis" OR review OR meta-analyses)

**EMBASE**

'appendicitis'/exp OR appendicitis OR (inflamma* AND ('appendix'/exp OR appendix)) OR appendiceal OR appendectom* OR appendicectom*

AND ('double blind procedure'/de OR 'meta analysis'/de OR 'meta analysis topic'/de OR 'randomized controlled trial'/de OR 'randomized controlled trial topic'/de OR 'systematic review'/de) OR random*

**CENTRAL**

(appendicitis OR (inflamma* AND appendix) OR appendiceal OR appendectom* OR appendicectom*):ti,ab,kw

OR MeSH descriptor: [Appendectomy] explode all trees
